# Editorial: New insights in microbial stress tolerance mechanisms

**DOI:** 10.3389/fmicb.2024.1513485

**Published:** 2024-12-10

**Authors:** Zeynep Petek Çakar, Hector Alex Saka, Jose Echenique

**Affiliations:** ^1^Department of Molecular Biology and Genetics, Faculty of Science and Letters, Istanbul Technical University, Istanbul, Türkiye; ^2^Dr. Orhan Ocalgiray Molecular Biology, Biotechnology and Genetics Research Center (ITU-MOBGAM), Istanbul Technical University, Istanbul, Türkiye; ^3^Centro de Investigaciones en Bioquímica Clínica e Inmunología (CIBICI)-Consejo Nacional de Investigaciones Científicas y Técnicas (CONICET), Córdoba, Argentina; ^4^Departamento de Bioquímica Clínica, Facultad de Ciencias Químicas, Universidad Nacional de Córdoba, Córdoba, Argentina

**Keywords:** bacteria, oxidative stress, metal stress, influenza A virus, adaptive laboratory evolution, fungi, salt stress, microbial stress response

In nature, microorganisms must survive a variety of stressors, including toxic metals, oxidative environments, pH changes, nutrient deprivation, antimicrobial agents, and host immune responses. This Research Topic comprises 14 manuscripts that explore different aspects of microbial stress tolerance mechanisms, with a particular emphasis on responses to metal, acidic, and oxidative stress, including studies on bacteria and filamentous fungi relevant for human health and also for biotechnological applications.

Heavy metal pollution is a major health and environmental issue. Chen et al. investigated the effects of chromium on the gut microbiota of silkworm and their metabolome. They identified a complex network emphasizing the interrelation between diverse bacterial species, their metabolic byproducts and functional significance during chromium stress. The authors suggested that this innovative approach, which combines metagenomic sequencing coupled with non-targeted metabolomics, can enhance our understanding of gut microbiota-metabolite interactions under chromium stress. This methodology may be useful for future investigations related to heavy metal-induced ecological disruption.

Metal(loid)s are toxic to microbial cells by generating reactive oxygen species (ROS). Through proteomic analysis and a genome-wide screening deletion collection of *Escherichia coli*, Cornejo et al. showed that certain metal(loid)s induce bacterial death by promoting the aggregation of specific proteins in a ROS-independent manner. The aggregated proteins were associated with several essential processes, including amino acid biosynthesis as enzymes. The authors proposed that metal(loid) toxicity is a multifactorial phenomenon that could lead to cell death.

*Vibrio cholerae* is an aquatic bacterium and the cause of cholera pandemics. Little is known about its response to stresses induced by heavy metals. Zhang B. et al. explored how *V. cholerae* responds to stress triggered by metals and found that Cd^2+^, Pb^2+^, and Zn^2+^ decreased bacterial cell membrane fluidity, while that of Ni^2+^ increased it. Surprisingly, these stressing stimuli increased the production of proteins related to adhesion, invasion, cell-damage and virulence, raising intriguing questions about their impact on *V. cholerae*-host interactions.

With their high metabolic versatility, *Rhodobacter* species are used to study photosynthesis, hydrogen production, bioremediation and biosensing. Atay et al. developed a robust and highly cobalt-stress-resistant *Rhodobacter sphaeroides* strain using adaptive laboratory evolution. The evolved strain was also resistant to other metal ions and salt stress, and could hold Co^2+^ ions, indicating its bioremediation potential. Genomic analyses revealed mutations in genes related to transcriptional regulators, NifB-family-FeMo-cofactor biosynthesis, putative virulence factors, TRAP-T family transporter, sodium/proton antiporter (NhaD), and genes with unknown functions that may be important in cobalt-stress-resistance.

Zamal et al. investigated the photosynthetic apparatus of a *Rhodobacter alkalitolerans* strain newly isolated from an alkaline pond, under high light intensity and alkalinity stress conditions. Although increased light intensity led to a decrease in the stability of the photosystem complexes at normal pH, acclimation to increased light intensity was observed in *R. alkalitolerans* at alkaline conditions, manifested as photoprotection at high pH. Based on increased expression of the antiporter NhaD, they suggested that it may be crucial in maintaining homeostatic balance in alkaline conditions.

*Streptococcus pneumoniae*, a bacterial human pathogen, must overcome different conditions to survive during infection, such as nutrient depletion, acidic, and oxidative stress. Zhang C. et al. described that methionine semi-starvation resulted in intracellular acidification and a subsequent bacterial growth retardation. Curiously, the addition of glutamine restored optimal intracellular pH and promoted pneumococcal growth, a correction attributed to glutamine deamination. The authors proposed that this novel adaptation mechanism for nutrient deficiency could provide new drug targets for inhibiting pneumococcal infections.

*S. pneumoniae* produces high levels of H_2_O_2_ to eliminate other microorganisms from the respiratory tract microbiota. Hernandez-Morfa, Olivero et al. provide a comprehensive analysis of the various strategies employed by this pathogen to counteract oxidative stress generated both by itself and by host cells, including the use of H_2_O_2_ scavengers. This review also focused on the relevance of metal homeostasis in the oxidative stress response regulation. In addition, a particular focus was given to the role of the oxidative stress response during the transient intracellular life of *S. pneumoniae*.

Hernandez-Morfa, Reinoso-Vizcaino et al. elucidated the impact of influenza A coinfection on the induction of fluoroquinolone persistence in *S. pneumoniae*. Viral infection increases the intracellular ROS production in host cells, which contributes to increased fluoroquinolone persistence. This enhancement is partially attributable to ROS because this phenotype manifests only in autophagy-proficient cells. The authors propose a novel mechanism by which viral infection promotes antibiotic persistence of *S. pneumoniae* within host cells, generating concern for the fluoroquinolone treatment of pneumococcal infections in patients with influenza.

Intracellular oxidative stress from H_2_O_2_/superoxide has been thought to potentially result in the oxidation of cysteine residues of cytoplasmic proteins. *E. coli* responds to H_2_O_2_ by inducing glutaredoxin-1 and thioredoxin-2, which seems to support that view. Eben and Imlay tested the abilities of different oxygen species to oxidize either model thiols or protein cysteine residues *in vitro*. They showed that chemical cysteine oxidations were rare events and cellular glutaredoxin and thioredoxin may have undiscovered roles under other growth conditions than those employed in their study.

*Pseudomonas aeruginosa* is a ubiquitous bacterium in soil and aquatic environments and a recognized antibiotic multi-resistant pathogen, especially within hospital settings. Sodium hypochlorite (NaOCl) is among the most effective disinfectants due to its potent oxidizing properties. da Cruz Nizer et al. investigated novel mechanisms employed by *P. aeruginosa* to combat NaOCl oxidative stress. Using transposon mutagenesis, they identified mutants with heightened susceptibility to NaOCl. Unexpectedly, this study revealed that *P. aeruginosa* leverages HCN production to mitigate NaOCl toxicity.

*Haemophilus influenzae*, a human bacterial pathogen, is able to survive in the presence of hypochlorite (OCl^−^) generated by immune cells. Nasreen et al. reported that the expression of the DmsABC S-/N-oxide reductase is induced by OCl^−^ and it is essential for bacterial survival during infection. They proposed that MsrAB methionine sulfoxide reductase is necessary for physical resistance to HOCl, DmsABC is essential for intracellular colonization, and MtsZ S-oxide reductase contributes to resistance against N-chlorotaurine. This study underscores the significance of these enzymes in bacterial virulence.

*Trichoderma harzianum* is a soil fungus used in agriculture as a biocontrol agent. However, its efficacy is limited by soil salinization and excessive pesticide use. Yang et al. carried out a detailed analysis of the transcriptional responses of *T. harzianum* to NaCl-induced stress revealing that genes involved in cellular detoxification, glutathione metabolism and active oxygen clearance were differentially expressed. Overall, this study provides significant insights into *T. harzianum'*s adaptation to salt stress, potentially improving biocontrol strategies.

*Aspergillus* species are fungi with biotechnological and ecological importance, and pathogenicity. As their genome structure and function are highly conserved, Jorge et al. tested if they also have conserved stress regulators. They integrated transcriptome signatures of different *Aspergillus* species to various organic compounds and identified a single gene, *AN9181*, assigned as NmrB, that showed the same response across different datasets. Comparison of the single deletion mutant Δ*AN9181* with the wild-type strain revealed that NmrB negatively regulates *Aspergillus nidulans* metabolism under oxidative stress condition.

*Listeria monocytogenes*, a foodborne pathogen that causes listeriosis in humans, is able to survive in different stressful environments. Ładziak et al. demonstrated that Lmo0946 is an SOS response interfering factor essential for normal growth in both stress-free and multi-stress conditions. In addition, Lmo0946 contributes to biofilm formation, susceptibility to β-lactam antibiotics, and virulence. The *lmo0946* mutation resulted in the induction of the SOS response, the mobilization of genetic elements, and the downregulation of genes related to bacterial general stress response and virulence.

In conclusion, this Research Topic provides novel insights into the molecular, biochemical, and physiological aspects of microbial stress response and tolerance strategies to survive in diverse environments, including soil, water, and host tissues. The diversity and complexity of stress response and tolerance mechanisms, particularly against heavy metal and oxidative stress, highlights the need for further investigations, using powerful strategies including adaptive laboratory evolution, omics analysis, and reverse engineering. Future research should delve deeper into the molecular mechanisms of protein aggregation and metal(loid)s-induced bacterial and fungal death. In addition, exploring strategies of pathogenic bacteria for survival within host cells and the impact of co-infection on microbial persistence and drug resistance could provide valuable therapeutic targets. Finally, elucidating the genetic and biochemical mechanisms regulating microbial response and tolerance to heavy metals, oxidative stress, antimicrobial drugs, and host immunity remains crucial for future advancements and innovative solutions in bioremediation and infectious disease control.

